# Phlebotomine sand flies (Diptera: Psychodidae, Phlebotominae) fauna in Unaí, State of Minas Gerais, Brazil

**DOI:** 10.1590/0037-8682-0290-2025

**Published:** 2026-04-17

**Authors:** Gabriele Zaine Teixeira Debortoli, Victor Luiz Gomes Batista, Fernanda Batista Santos, Lucas Mendes Diniz, Pedro Augusto Batista Silva, Valentine Tavares Ferreira, Carlos Augusto dos Santos Sousa, Katia Denise Saraiva Bresciani, Ricardo Andrade Barata, Thaís Rabelo Santos-Doni

**Affiliations:** 1 Universidade Federal dos Vales do Jequitinhonha e Mucuri, Programa de Pós-graduação em Biologia Animal, Diamantina, MG, Brasil.; 2 Universidade Federal de Minas Gerais, Programa de Pós-graduação em Parasitologia, Belo Horizonte, MG, Brasil.; 3 Gerência Regional de Saúde de Unaí, Unaí, MG, Brasil.; 4 Universidade Federal dos Vales do Jequitinhonha e Mucuri, Instituto de Ciências Agrárias, Unaí, MG, Brasil.; 5 Universidade Estadual Paulista, Faculdade de Medicina Veterinária, Araçatuba, SP, Brasil.

**Keywords:** Lutzomyia, Biodiversity, Leishmaniases, Insect vector, Spatial distribution, Climate

## Abstract

**Background:**

Leishmaniasis, a neglected disease caused by *Leishmania* protozoa, is transmitted by phlebotomine sand flies and has a significant public health impact. The municipality of Unaí, Minas Gerais, reports human and canine cases of leishmaniasis; however, entomological data remain limited. This study aimed to describe the local phlebotomine sand fly fauna and environmental and climatic factors influencing its occurrence.

**Methods:**

Sampling was conducted monthly from January to December 2024 in ten households distributed across seven neighborhoods of Unaí. HP light traps were installed in both intradomestic and peridomestic regions. The captured specimens were morphologically identified and statistically analyzed to assess their association with environmental and climatic variables using mixed-effects linear models.

**Results:**

Overall, 504 phlebotomine sand flies, representing 11 species, were captured. *Lutzomyia longipalpis* (39.7%) was the most abundant species, followed by *Ev. lenti* (33.3%) and *Ny. intermedia* (15.3%). The peridomestic areas exhibited greater species diversity and abundance, particularly in the presence of chicken coops, organic matter, and vegetation. Temperature is positively associated with *Lu. longipalpis* and *Ev. lenti*, whereas precipitation negatively affected *Ny. intermedia*. Males comprised 69.8% of captured specimens, suggesting the presence of active breeding sites near the households.

**Conclusions:**

This study provides the first record of the phlebotomine sand fly fauna in Unaí. Environmental conditions favor vector species in settings with close human-animal interactions, increasing the risk of domiciliary transmission. These findings highlight the importance of sustained entomological surveillance and environmental management measures as complementary strategies to control leishmaniasis.

## INTRODUCTION

Leishmaniasis comprises a group of neglected diseases caused by protozoa of the genus *Leishmania* (Kinetoplastida: Trypanosomatidae), which is transmitted primarily through the bite of infected female phlebotomine sand flies (Diptera: Psychodidae: Phlebotominae)^1^ ^,^ ^2^. According to the World Health Organization (WHO), more than one billion people reside in endemic areas and are at risk of infection. Clinically, Leishmaniasis manifests in three main forms: visceral (VL), cutaneous (CL), and mucocutaneous. Visceral leishmaniasis, caused by *Leishmania infantum*, is considered endemic to several regions of Brazil, accounting for approximately 94% of the reported cases in the Americas^3^ ^,^ ^4^.

Globally, 1,067 sand fly species (Phlebotominae) have been recognized^5^ ^,^ ^6^, of which 557 are recorded in the Americas. Brazil harbors the greatest diversity of sandfly species, with 300 species recorded nationally, including 98 species in the state of Minas Gerais^7^ ^,^ ^8^. *Lutzomyia longipalpis*, the principal vector of visceral leishmaniasis in Brazil, is widely distributed throughout South America and exhibits marked ecological plasticity, particularly in urban and peri-urban environments^9^. Reflecting its broad distribution, the FIOCRUZ-COLFLEB catalog, one of the largest sand fly collections worldwide, includes 3,682 records of *Lu. longipalpis* across the country^10^ ^,^ ^11^.

Phlebotomine sand flies are present in all Brazilian federal units, reflecting their remarkable adaptability to diverse environmental conditions^12^. These insects are crepuscular and nocturnal and their hematophagous females feed on a wide variety of vertebrate hosts, including wild and domestic mammals^13^. Their abundance and spatial distribution are strongly influenced by several environmental factors, such as the presence of decomposing organic matter, chicken coops, and climatic variables including temperature, humidity, precipitation, and wind speed^14^ ^,^ ^15^. 

Despite the documented occurrence of human and animal cases of both VL and CL in Unaí, Minas Gerais (Brazil), with 16 confirmed cases of human visceral leishmaniasis and 228 cases of human cutaneous leishmaniasis recorded in the national SINAN/DATASUS database between 2014 and 2024^16^, no studies have systematically conducted entomological surveys of the local sand fly fauna. The lack of baseline entomological data hinders the comprehensive understanding of local transmission dynamics and limits the development of effective surveillance and vector control strategies.

## METHODS

### Study area

The study was conducted in the municipality of Unaí (16°22′45″S, 46°53′45″W), state of Minas Gerais, southeastern Brazil. The municipality encompasses an area of 8,445.432 km², with an administrative center situated at an altitude of 640 m above sea level^17^. The topography is predominantly flat and lies within the São Francisco River basin. The climate is classified as humid tropical, with a mean annual relative humidity of 63.3%, and annual temperatures of 24°C ranging from 10 to 35°C. The mean annual precipitation is approximately 1,200 mm, with the rainy season extending from October to March. 

### Ethical issues

In this study, all procedures complied with the Ethical Principles in Animal Research adopted by the College of Animal Experimentation and were approved (protocol number PP 007/2024) by the Ethical Committee for Animal Welfare, IPESA, Minas Gerais state, Brazil.

### Sand fly trapping

Households were selected based on three main criteria: a history of reported human or canine VL cases within the last 2 years, provided by the Municipal Health Department and the Zoonosis Control Center of Unaí; the presence of at least two of the following environmental features: chicken coop, pigsty, fruit trees, or accumulated organic matter; and owner consent. Sample collection was performed in ten households distributed across seven neighborhoods. A total of 20 HP light traps, described in detail by Pugedo et al.^18^ were deployed over two consecutive nights from 6:00 p.m. to 6:00 a.m. during the last week of each month from January to December 2024. The total sampling effort amounted to 20 traps per night × 2 nights per month × 12 months = 480 trap nights. Two traps were installed in each household: one in the intradomestic ecotope inside the house and the other in the peridomestic ecotope, up to 100 m from the house. 

### Morphological identification of sand flies

Sandfly samples were preserved in 70% ethanol and processed at the Animal Parasitology Laboratory of the Federal University of Jequitinhonha and Mucuri Valleys. Specimens were prepared according to the protocol described by Langeron^19^. Species identification was performed based on morphological characteristics, using the taxonomic key proposed by Galati^20^. The structures analyzed as part of the diagnosis included the head (antennae, cibarium, pharynx, and palps), thorax (wings and legs), and abdomen (external and internal genitalia). For female specimens, the last two abdominal segments were carefully dissected to allow visualization of the internal genitalia, thus minimizing the risk of damage to the spermathecae.

### Sand fly mapping and distribution

**FIGURE 1:**
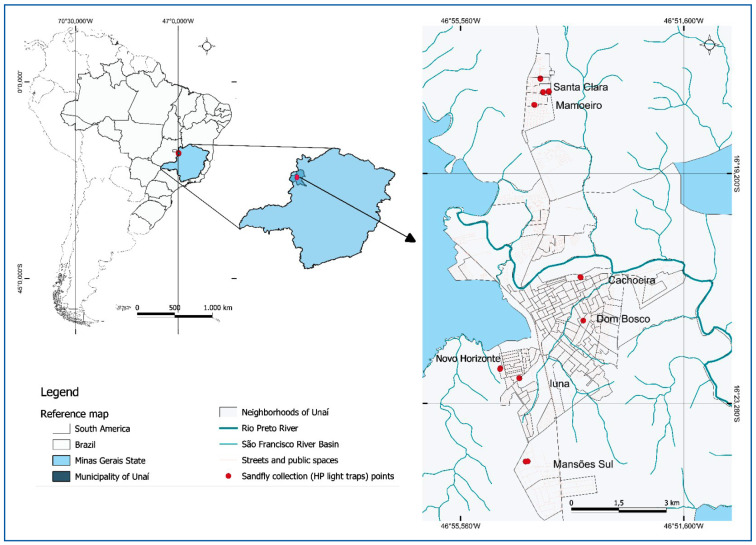
Sand Fly Collection Sites in the Municipality of Unaí, Minas Gerais, Brazil.

Spatial analysis of phlebotomine abundance was performed using the Geographic Information System software QGIS® 3.26 Buenos Aires (QGIS Development Team, 2022). The municipality of Unaí, Minas Gerais, was georeferenced **(** Figure 1 **)**, including the locations of street layouts, river basins, and the locations of households where HP light traps were installed for sand fly collection. The selected households were distributed across seven neighborhoods with different environmental and urbanization characteristics: Cachoeira, Dom Bosco, Iuna, Mamoeiro, Mansões Sul, Novo Horizonte, and Santa Clara. Based on these data, a kernel density map was generated to represent the spatial concentration of vectors across the municipality census sectors. Additionally, buffer zones of 1.5 km radius were established around each sampling site to indicate the potential areas of phlebotomine influence.

### Environmental factors and their sources

The meteorological data used in this study were obtained from the online portal of the National Institute of Meteorology (INMET). Four predictive climatic variables were analyzed: temperature, relative humidity, wind speed, and precipitation. Data were derived from daily records at Unai Conventional Meteorological Station 83428, Minas Gerais. Data were compiled as monthly averages for analysis based on the information available in the INMET database.

### Calculation of sand fly diversity values

To assess the species diversity across neighborhoods, the Shannon diversity index was calculated using the following formula:

$$H=\;-\sum \left[\left(pi\right)\;.\log\left(pi\right)\right]$$

Where pᵢ is the proportion of individuals of species i in relation to the total number of individuals in the sample. This index considers the number of species and the evenness of their relative abundance. Additionally, Simpson’s diversity index was used to estimate the probability that two individuals randomly selected from a sample were likely to belong to different species. The index is calculated using the following formula:

$$D=\sum_{i=1}^{s}\frac{n_{i}(n_{i}-1)}{N(N-1)}$$

Where nᵢ is the number of organisms of a particular species and N is the number of organisms of all species^21^. 

### Statistical analysis

Descriptive statistics were used to summarize the abundance of phlebotomine sand flies and climatic variables, including the arithmetic mean, standard deviation, median, minimum, and maximum values. The normality of the continuous data was assessed using visual inspection and statistical criteria. To compare the differences in climatic conditions between months, a one-way analysis of variance (ANOVA) was applied to evaluate the probability of significance (p-value) for F-statistics.

To assess the association between the presence of sand fly species (dependent variable) and environmental and biological predictors (independent variables), Generalized Linear Mixed Models (GLMMs) were fitted using a binomial distribution and logit link function. Separate models were developed for *Lu. longipalpis*, *Evandromyia lenti*, and *Nyssomyia intermedia*. The fixed effects included the area of capture (intradomestic or peridomestic), sex of the insect, average temperature (on the day of capture and for the previous 7, 15, and 30 days), average humidity, wind speed, and accumulated precipitation across the same periods. Neighborhoods and houses were included as nested random effects to account for clustering.

In the first stage, univariate analyses were conducted to screen for variables potentially associated with outcomes. Variables with p-values ≤ 0.25 were selected for inclusion in multivariate models. Collinearity among the predictors was assessed using the Variance Inflation Factor (VIF), and highly correlated variables were excluded to avoid multicollinearity. The final multivariate models were obtained by sequentially excluding non-significant variables while monitoring model changes, fit, and effect estimates.

All models were fitted in Stata® version 16.1 (StataCorp LLC, College Station, TX, USA), and odds ratios (OR) and adjusted odds ratios (AOR) were presented with 95% confidence intervals. The level of statistical significance was set at 5%.

## RESULTS

Table 1 highlights the main characteristics of the domestic environments of the ten households included in the study, which are known to frequently attract and shelter phlebotomine sand flies. The primary differences among these locations involved the size of the outdoor area, presence of fruit trees, chicken coops, domestic animals, organic matter, and waste. The neighborhoods of Cachoeira (C6), Mamoeiro (C3), and Mansões Sul (C9) had more vegetation in peridomestic areas, with greater shade and a higher number of animals, especially chickens (> 15). Household C3 harbored the highest diversity of domestic animal species, including dogs (n=3), cats (n=2), horses (n=3), one calf, and chickens (>15). In total, chicken coops were recorded in eight of the ten households, were more common in the peripheral areas. 

**TABLE 1:** Description of the peridomestic environment and presence of domesticated animals in sampled households across neighborhoods of Unaí, Minas Gerais, Brazil.

Neighborhoods	House	General characteristics	Domesticated animals (number)
Cachoeira	C6	Located in the northeastern periphery of the city.	Dogs (1); Chickens (>15); Pigs (1)
		Extensive peridomestic area with medium to large trees, some of them fruit-bearing, abundant organic matter, and high humidity.	
		Collection point closest to the Rio Preto River (approximately 665 meters away).	
Dom Bosco	C1	Located in a residential neighborhood near the city center.	Dogs (4); Cats (3)
		Two medium-sized fruit trees.	
		Predominantly urbanized environment, with a smaller peridomestic area compared to the other sites.	
Iuna	C10	Located in the southwestern periphery of the city.	Dogs (1); Chickens (<15); Cats (2)
		Chicken coop and presence of domestic animals.	
Mamoeiro	C3	Located in the northern periphery of the city	Dogs (3); Cats (2); Horses (3); Calves (1); Chickens (>15)
		Peridomestic area adjacent to a well-preserved expanse of residual Cerrado, rich in organic matter, humidity, and with the presence of domestic animals and wild mammals.	
		Presence of some fruit trees and household waste in the yard.	
	C4	Located in the northern periphery of the city	
		Extensive peridomestic area with medium to large trees, some of them fruit-bearing.	
	C7	Located in the northern periphery of the city	Dogs (2); Chickens (>15)
		Presence of a chicken coop and dogs, with a dirt yard rich in organic matter.	
	C8	Located in the northern periphery of the city	Dogs (2); Chickens (<15)
		Chicken coop and presence of organic matter.	
Mansões Sul	C9	Located in the southern periphery of the city.	Dogs (2); Chickens (>15)
		Largest peridomestic area compared to the other sites; medium and large trees, some of them fruit-bearing. Presence of a chicken coop.	
Novo Horizonte	C2	Located in the southwestern periphery of the city.	
		Peridomestic area bordered by native vegetation and small to medium-sized trees (some fruit-bearing), with abundant organic matter and humidity. Open sewage and stormwater drainage are located 30 meters away.	
Santa Clara	C5	Located in the northern periphery of the city	Dogs (1); Chickens (<15)
		Extensive peridomestic area with medium to large trees, some of them fruit-bearing.	
		Abundant organic matter in the yard and small chicken coop.	

The influence of these environmental characteristics on sandfly populations was reflected in the species composition and abundance observed across the sampled neighborhoods. Between January and December 2024, a total of 504 phlebotomine sand fly specimens were captured, representing 11 species, across 10 households distributed among seven neighborhoods in the municipality of Unaí, Minas Gerais (Table 2). A total of 352 males (69.8%) and 152 females (30.2%) were included with an overall mean density of 0.98 specimens/trap-night.

**TABLE 2:** Sand fly species composition and abundance in seven neighborhoods in Unaí, Minas Gerais, Brazil.

	Cachoeira			Dom Bosco			Iuna			Mamoeiro			Mansões Sul			Novo Horizonte			Santa Clara			Total			RA*	p-value**
	M	F	Total	M	F	Total	M	F	Total	M	F	Total	M	F	Total	M	F	Total	M	F	Total	M	F	Total		
*Lutzomyia longipalpis*	48	9	57	0	1	1	3	0	3	77	18	95	33	4	37	4	2	6	0	1	1	165	35	200	39.7	0.01
*Evandromyia lenti*	16	10	26	0	0	0	0	0	0	83	50	133	3	2	5	0	1	1	1	2	3	103	65	168	33.3	< 0.0001
*Nyssomyia intermedia*	36	8	44	0	0	0	0	0	0	8	7	15	7	2	9	1	8	9	0	0	0	52	25	77	15.3	< 0.0001
*Nyssomyia whitmani*	5	0	5	0	0	0	0	0	0	8	1	9	3	3	6	0	3	3	0	0	0	16	7	23	4.6	0.245
*Evandromyia sallesi*	1	0	1	0	0	0	0	0	0	2	0	2	2	1	3	0	2	2	0	0	0	5	3	8	1.6	0.059
*Evandromyia evandroi*	0	1	1	0	0	0	0	0	0	3	2	5	0	0	0	0	0	0	0	0	0	3	3	6	1.2	0.868
*Lutzomyia* sp*.*	0	1	1	0	0	0	0	0	0	0	4	4	0	0	0	0	0	0	0	0	0	0	5	5	1.0	0.938
*Psathyromyia termitophila*	1	0	1	0	0	0	0	1	1	0	2	2	0	0	0	1	0	1	0	0	0	2	3	5	1.0	< 0.01
*Psathyromyia sordellii*	0	0	0	0	0	0	0	0	0	0	1	1	1	0	1	1	1	2	0	0	0	2	2	4	0.8	< 0.01
*Psathyromyia lutziana*	0	0	0	0	0	0	0	1	1	0	0	0	0	2	2	0	0	0	0	0	0	0	3	3	0.6	< 0.001
Not identified	0	0	0	0	0	0	0	0	0	1	0	1	0	0	0	0	0	0	0	0	0	1	0	1	0.4	-
*Brumptomyia* sp*.*	0	0	0	0	0	0	0	0	0	0	0	0	0	0	0	0	1	1	0	0	0	0	1	1	0.2	< 0.01
*Psathyromyia quinquefer*	1	0	1	0	0	0	0	0	0	0	0	0	0	0	0	0	0	0	0	0	0	1	0	1	0.2	0.848
*Nyssomyia* sp.	0	0	0	0	0	0	0	0	0	0	0	0	1	0	1	0	0	0	0	0	0	1	0	1	0.2	0.333
Total	137			1			5			268			64			25			4			352	152	504		
Trap-nights	36			36			36			144			36			36			36			360				
Density (average no.) of sand flies	3.81			0.03			0.14			1.86			1.78			0.69			0.11			0.98				
Minimum	0			0			0			0			0			0			0			1				
Maximum	57			1			3			133			37			9			3			200				
Species richness	7			1			3			8			8			7			2			11				
Species diversity																										
Shannon-Wiener Index	1.31			0			0.95			1.16			1.40			1.63			0.56			1.43				
Simpson Index	0.69			0			0.70			0.61			0.64			0.80			0.50			0.70				

**M:** male; **F:** female; RA*: Relative abundance. ** Kruskal-Wallis test (Neighborhoods).

*Lutzomyia longipalpis* was the predominant species (39.7%), followed by *Ev. lenti* (33.3%) and *Ny. intermedia* (15.3%). The three most frequent species exhibited statistically significant differences among neighborhoods (*Lu. longipalpis*, p = 0.01; *Ev. lenti*, p < 0.0001; *Ny. intermedia*, p < 0.0001).

Among the neighborhoods, Mamoeiro had the highest absolute number of phlebotomine sand flies (n = 268; 53.2% of the total), followed by Cachoeira (n = 137; 27.2%), which also exhibited the highest capture density (3.81 specimens/trap-night), likely due to the combined presence of a chicken coop, pigsty, and dense peridomestic vegetation. In contrast, Dom Bosco (n = 1) and Santa Clara (n = 4) exhibited the lowest capture rates.

Species diversity complemented the observed patterns of species abundance, which varied substantially among neighborhoods. Assessed by the Shannon diversity index (H'), the highest indices were observed in Novo Horizonte (H' = 1.6), Mansões Sul (H' = 1.4), and Cachoeira (H' = 1.3), while the lowest were recorded in Dom Bosco (H' = 0) and Santa Clara (H' = 0.6). These indices were presented as descriptive measures of community diversity.

**FIGURE 2:**
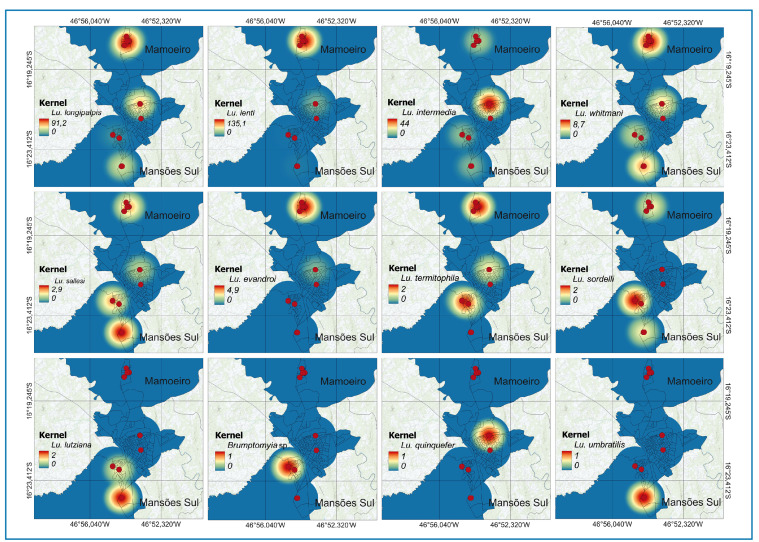
Spatial distribution and abundance intensity (Kernel density) of phlebotomine sand fly species captured in Unaí, Minas Gerais, Brazil.

The spatial distribution of phlebotomine species across the sampled neighborhoods is shown in kernel density maps **(** Figure 2 **)**. *Lutzomyia longipalpis*, *Ev. lenti*, and *Ny. intermedia* exhibited the highest spatial densities, with hotspots primarily concentrated in the Mamoeiro neighborhood.

In addition to environmental characteristics, climatic variables were analyzed for their potential influence on sand fly abundance. For each sampling event, the climatic averages were calculated based on the number of days when the traps were installed and for the preceding 7, 15, and 30 days. The monthly climatic variables recorded between January and December 2024 showed no statistically significant differences (p > 0.05). 

Mean values for temperature (26.9°C to 27.3°C), relative humidity (60.4% to 67.5%), mean wind speed (1.0 to 1.1 m/s), and accumulated precipitation (0.0 to 3.5 mm) showed limited variation across the periods analyzed, with no statistically significant differences (p > 0.05). The climatic variables remained relatively stable throughout the study period, indicating no direct influence on the abundance of phlebotomine sand flies in the sampled areas. The influence of these climatic and environmental factors on the abundance of the main species was further explored using a mixed-effects linear regression analysis (Table 3). The analysis revealed that the abundance of *Lu. longipalpis* was significantly higher in peridomestic areas (AOR = 2.01; p = 0.01) and among males (AOR = 3.12; p < 0.0001). Among the climatic variables, mean temperature over the previous 15 days was positively associated with the abundance of *Lu. longipalpis* (AOR = 2.03; p < 0.0001), whereas the mean temperature over the previous 30 d was negatively associated (AOR = 0.42; p < 0.0001). No significant associations were observed between humidity, wind speed, or precipitation.

**TABLE 3:** Variation in phlebotomine abundance analyzed using Generalized Linear Mixed Models (binomial family; logit link), with neighborhoods and houses included as a random effect. Unaí, Minas Gerais, Brazil.

Variables	OR*	95% Confidence Interval	SE**	Z statistic	p-value	AOR***	95% Confidence Interval	p-value
***Lutzomyia longipalpis***								
Area (Intra reference)	2.03	(1.21-3.41)	0.53	2.68	0.01	2.01	(1.16-3.48)	0.01
Sex (Female reference)	3.00	(1.84-4.90)	0.08	4.38	< 0.0001	3.12	(1.87-5.21)	< 0.0001
**Temperature average (ºC)**								
Collections days	1.07	(0.97-1.19)	0.05	1.43	0.15			
Previous 7	0.99	(0.84-1.16)	0.08	-0.17	0.87			
Previous 15	1.14	( 0.90-1.44)	0.14	1.15	0.25	2.03	(1.44-2.88)	< 0.0001
Previous 30	0.74	( 0.59-0.93)	0.09	-2.59	0.01	0.42	(0.28-0.61)	< 0.0001
**Humidity average (%)**								
Collections days	0.99	(0.97-1.01)	0.01	-1.40	0.16			
Previous 7	0.99	(0.97-1.01)	0.01	-0.82	0.42			
Previous 15	0.99	(0.96-1.01)	0.01	-0.95	0.34			
Previous 30	1.02	(0.99-1.05)	0.02	1.42	0.16	1.02	(0.97-1.06)	0.32
**Wind speed average (km/h)**								
Collections days	0.64	(0.41-0.98)	0.14	-2.04	0.04			
Previous 7	1.60	(1.00- 2.57)	0.38	1.97	0.05	1.10	(0.64-1.89)	0.74
Previous 15	0.33	(0.16-0.70)	0.13	-2.90	0.01			
Previous 30	0.28	(0.11-0.69)	0.13	-2.78	0.01			
**Accumulated precipitation (mm³)**								
Collections days	1.00	(0.96-1.04)	0.02	-0.17	0.86			
Previous 7	1.04	(1.00-1.07)	0.02	2.00	0.05	0.60	(0.28-1.27)	0.18
Previous 15	1.04	(1.00-1.09)	0.03	1.64	0.10			
Previous 30	1.01	(0.96-1.07)	0.04	0.49	0.63			
***Lutzomyia lenti***								
Area (Peri reference)	3.71	(2.21-6.20)	0.97	5.00	< 0.0001	3.82	(2.19-6.67)	< 0.0001
Sex (Male reference)	1.87	(1.14-3.06)	0.47	2.47	0.01	1.76	( 1.03-3.01)	0.04
**Temperature average (ºC)**								
Collections days	0.86	(0.77-0.96)	0.05	-2.82	0.01			
Previous 7	0.99	(0.82-1.19)	0.09	-0.10	0.92			
Previous 15	0.78	(0.60-1.02)	0.11	-1.81	0.07	0.64	(0.46-0.90)	0.01
Previous 30	1.47	(1.16-1.87)	0.18	3.15	< 0.001	1.71	(1.24-2.36)	0.001
**Humidity average (%)**								
Collections days	1.02	(1.00-1.04)	0.01	1.73	0.08			
Previous 7	1.00	(0.97-1.03)	0.01	-0.04	0.97			
Previous 15	1.00	(0.97-1.03)	0.02	-0.27	0.79			
Previous 30	0.94	(0.91-0.97)	0.02	-3.69	<0.0001	0.97	(0.93-1.02)	0.22
**Wind speed average (m/s)**								
Collections days	1.45	(0.91-2.31)	0.34	1.58	0.12	1.15	(0.60-2.23)	0.68
Previous 7	0.45	(0.27-0.74)	0.12	-3.09	< 0.001	0.85	(0.38-1.89)	0.69
Previous 15	4.08	(1.36-12.27)	2.29	2.50	0.01			
Previous 30	5.56	(1.46-21.19)	3.79	2.51	0.01			
**Accumulated precipitation (mm³)**								
Collections days	1.03	(0.98-1.08)	0.03	1.25	0.21			
Previous 7	0.93	(0.89-0.97)	0.02	-3.48	< 0.0001	0.98	(0.92-1.05)	0.51
Previous 15	0.93	(0.89-0.97)	0.03	-3.08	< 0.001			
Previous 30	0.93	(0.88-0.99)	0.03	-3.08	< 0.001			
***Lutzomyia intermedia***								
Area (Intra reference)	3.35	(1.45-7.78)	1.44	2.82	0.01	3.04	(1.30-7.13)	0.01
Sex (Female reference)	1.25	(0.70-2.23)	0.37	0.75	0.45	1.45	(0.79-2.65)	0.24
**Temperature average (ºC)**								
Collections days	1.14	(1.00-1.30)	0.08	1.97	0.05	0.80	(0.57-1.12)	0.19
Previous 7	1.19	(0.97-1.46)	0.12	1.65	0.10			
Previous 15	1.26	(0.93 -1.70)	0.20	1.47	0.14			
Previous 30	0.98	(0.73-1.33)	0.15	-0.10	0.92			
**Humidity average (%)**								
Collections days	0.99	(0.97-1.02)	0.01	-0.80	0.43			
Previous 7	1.00	(0.97-1.03)	0.02	0.05	0.96			
Previous 15	1.01	(0.97-1.05)	0.02	0.57	0.57			
Previous 30	1.05	(1.00-1.11)	0.03	1.94	0.05	1.08	(0.99-1.19)	0.09
**Wind speed average (km/h)**								
Collections days	1.53	(0.89-2.64)	0.43	1.54	0.13	0.84	(0.33-2.15)	0.72
Previous 7	1.39	(0.72-2.71)	0.47	0.97	0.33			
Previous 15	1.98	(0.43-3.33)	0.63	0.35	0.73			
Previous 30	1.02	(0.28-3.73)	0.67	0.04	0.97			
**Accumulated precipitation (mm³)**								
Collections days	0.95	(0.90-1.00)	0.03	-1.90	0.06	0.16	(0.03-0.80)	0.03
Previous 7	1.03	( 0.99-1.08)	0.02	1.29	0.20	2.21	(0.49-9.92)	0.30
Previous 15	1.04	(0.98-1.11)	0.03	1.28	0.20			
Previous 30	1.08	(1.01-1.15)	0.04	2.32	0.02			

* OR: odds ratio; ** SE: Standard error; *** AOR: adjusted odds ratio.

The abundance of *Ev. lenti* was significantly higher in the intradomestic areas (AOR = 3.82; p < 0.0001) and in females (AOR = 1.76; p = 0.040). Among the climatic variables, the mean temperature over the previous 30 days showed a positive association (AOR = 1.71; p = 0.001), whereas the mean temperature over the previous 15 days showed a negative association (AOR = 0.64; p = 0.010). No significant associations were observed between changes in humidity, wind speed, or precipitation and the abundance of *Ev. lenti*.

For *Ny. intermedia* was higher in peridomestic areas (AOR = 3.04; p = 0.010). No statistically significant association was observed between sex, although the odds ratio suggested a trend toward a higher abundance among males (AOR = 1.45; p = 0.235). Among the climatic variables, precipitation on the day of collection (AOR = 0.16; p = 0.026) was negatively correlated.

Temperature was the only climatic variable significantly associated with the abundance of *Lu. longipalpis* and *Ev. lenti*, while precipitation was associated with *Ny. intermedia* abundance, with distinct effects across species and at different temporal scales. No significant associations were observed between humidity and wind speed for the analyzed species.

## DISCUSSION

This study represents the first entomological survey conducted in Unaí, Minas Gerais, Brazil, offering novel insights into the species composition, spatial distribution, and climatic variables influencing sand fly populations in the region. Compared to studies from nearby municipalities in northwestern Minas Gerais, such as Paracatu^22^, our findings showed similar patterns of species composition and intra- and peridomestic distributions. These findings are particularly relevant given the high incidence of visceral leishmaniasis in Minas Gerais, and the relevance of entomological data for Unaí is reinforced by the occurrence of human cases without prior vector studies, emphasizing the public health importance of understanding vector ecology in endemic areas^23^ ^-^ ^25^.

The dynamics of leishmaniasis transmission are significantly influenced by the ecological plasticity of phlebotomine vectors, which enables their adaptation to anthropized and urban environments. Environmental degradation (deforestation) caused by agricultural expansion and other changes (migration, irregular land occupation, poor sanitation, and an increase in domestic animals in the peridomestic environment) has been associated with changes in the transmission patterns of leishmaniases^26^, favoring their establishment in peridomestic areas where they find shelter and food sources^27^ ^,^ ^28^.

In this study, the neighborhoods of Mamoeiro and Cachoeira recorded the highest sandfly abundance. Both sites presented typical peridomestic characteristics (e.g., presence of poultry coops, domestic animals, organic matter, and proximity to vegetation), which created favorable conditions for the presence and maintenance of phlebotomine populations^29^ ^-^ ^31^. Chicken coops and pigsties are recognized as resting sites for adult sandflies of both sexes and as feeding sites for females^32^ ^,^ ^33^. Moreover, these ecotopes provide shade, moisture, and soil rich in organic matter, offering suitable conditions as breeding sites for immature sand flies^34^ ^,^ ^35^. 

Several findings suggest an increased risk of domiciliary transmission of VL in Unai: (a) the predominance of captured male sand flies indicates the presence of active breeding sites in the areas surrounding the sampled households, (b) peridomestic areas revealed significantly higher species diversity and sand fly abundance than intradomestic environments, and (c) synanthropic species such as *Ny. intermedia* and *Nyssomyia whitmani* were more abundant in animal shelters in close proximity to human dwellings.

Peridomestic areas accounted for the highest species richness (11 species) and total abundance (324 individuals), which is consistent with findings from other Brazilian municipalities that have demonstrated similar ecological preferences^36^ ^,^ ^37^. 

*Lutzomyia longipalpis*, the primary vector of Le. infantum and the most abundant species in this study (39.7% of captures), was detected in all sampled neighborhoods. This corroborates its predominance over other species in areas where VL is endemic across Brazil^38^ ^-^ ^41^.

The second-most abundant vector species, *Ny. intermedia*, which is a known vector of *Le. braziliensis*, also showed a broad distribution across both intradomestic and peridomestic ecotopes in Unaí. Environmental changes resulting from anthropogenic activities may have contributed to the dominance of *Ny. intermedia*. Generalist feeding behavior of *Ny. intermedia* explains its higher occurrence in the peridomestic areas. Its higher reproductive potential in peridomestic environments is likely the cause of its high density and predominance over other species in this environment^42^. Other studies have also reported the predominance of *Ny. intermedia* in the anthropogenic or peridomestic areas^43^ ^-^ ^44^.

The probability of capturing *Lu. longipalpis* was 2.2 times higher in peridomestic areas than in intradomestic environments, whereas *Ny. intermedia*, this likelihood was 3.35 times more higher in these ectopes than in intradomestic sites. These findings highlight the preference of both species for transitional ecotopes characterized by vegetation, animal shelters, organic matter, and host availability.

Notably, *Lu. longipalpis* appear to be fully adapted to anthropogenic environments, as observed in other areas^28^.

In addition to the confirmed CL and VL vectors, other species identified in this study warrant epidemiological consideration. *Ev. lenti*, *Ev. sallesi* and *Sciopemyia sordellii* have been described as harboring *Leishmania* DNA, including *Le. braziliensis* and *Le. infantum* ^41^ ^,^ ^45^ ^-^ ^48^.

*Evandromyia lenti* was found approximately three times more frequently in intra-domestic environments than in peridomestic environments, echoing the urban patterns reported in other endemic regions with environmental and socioeconomic characteristics similar to those in the studied áreas^46^. This suggests that the species exhibits endophilic behavior, residing in habitats in proximity to suitable hosts. In Campo Grande, this species is closely associated with peridomestic areas^49^ and domestic shelters in the rural areas of Mato Grosso do Sul^50^. However, Brazil et al.^51^ reported *Ev. lenti* refractory to *Leishmania* infection, and the presence of *Le. infantum* DNA plays a potential role in the epidemiology of visceral leishmaniasis^46^ ^,^ ^48^ ^,^ ^52^.

Studies have indicated that the occurrence and seasonal fluctuation of sandfly populations can be modulated by climatic factors, such as temperature, relative humidity, precipitation, and wind speed, which influence their development, flight activity, and survival^53^ ^-^ ^55^.

*Lutzomyia longipalpis* was found throughout the sampling period, with high abundance during summer (n = 65) and autumn (n = 96), likely because of rainfall in the preceding seasons (October to January), which may have enhanced vegetation growth, reduced solar radiation, increased soil moisture, and consequently provided optimal conditions for insect development. A negative association was observed with prolonged dry periods, which are likely to result in arid conditions that hinder the development of both immature and adult stages, suggesting a definite thermal tolerance threshold.

Similar results were reported by Oliveira et al.^56^, who observed a high abundance of *Lu. longipalpis* after a one- to two-month lag in rainfall. Indirectly supporting this, there is evidence that the period of highest visceral leishmaniasis transmission occurs during and shortly after the rainy season, when the insect population density increases^57^.

This study also provided evidence of a positive association between *Lu. longipalpis* and precipitation and temperature (previous 15, AOR: 2.03), and a negative association (AOR: 0.42) with lagged temperature (previous 30), indicating that modest short-term increases in temperature further increase the likelihood of capturing the species. The negative association with temperature suggests a higher tolerance limit for this vector^33^ ^,^ ^58^, suggesting that prolonged heat may suppress population abundance, possibly owing to increased mortality or reduced activity at suboptimal thresholds.

Previous studies have demonstrated that temperature influences the dynamics of *Lu. longipalpis* ^53^. Martins et al.^59^ reported that female sand flies modulate their behavior in response to ambient temperature, exhibiting reduced activity at lower temperatures and increased host-seeking behavior under relatively warmer conditions as blood digestion progresses. 

*Nyssomyia intermedia* was consistently observed throughout the study period, with peaks during the warmer and wetter months (summer and autumn); similar results were reported by Virgens et al.^60^ and Condino et al.^61^. Unlike *Lu. longipalpis*, which responds to temperature variation, *Ny. intermedia* was primarily influenced by precipitation, reflecting the distinct ecological preferences of this species. Rainfall is a key environmental factor that influences the population dynamics of phlebotomine sand flies^62^. Precipitation on the day of collection was associated with a marked decrease in the occurrence of this species, possibly because of reduced flight activity during heavy rainfall^63^. 

Studies on the diversity and distribution of phlebotomine species have provided critical insights into the epidemiology of leishmaniasis^64^, and effectively guiding and implementing preventive measures and improving community health. Environmental management is a critical strategy for regulating the population density of *Lu. longipalpis*, in Brazil^65^.

A limitation of this study was the absence of molecular screening for *Leishmania* DNA in female specimens of *Lu. longipalpis*, *Ny. intermedia*, and *Ny. whitmani*, due to budget constraints. Future investigations should incorporate molecular analyses to estimate natural infection rates, which would enhance understanding of local transmission dynamics and support targeted control measures. 

In conclusion, this study provides the first record of sand fly fauna in Unaí, Minas Gerais, demonstrating the predominance of *Lu. longipalpis*, *Ev. lenti*, and *Ny. intermedia*. Peridomestic environmental characteristics significantly influence vector abundance and spatial distribution , while climatic variables, such as temperature and precipitation modulate their populations in species-specific and time-dependent manner. The abundance of vector species in environments with favorable breeding conditions and close human-animal contact reinforces the risk of domiciliary transmission of leishmaniases. Continued entomological surveillance, integrated with environmental management, is essential for effective vector control strategy. 

## References

[1] Ansari Z Chaurasia A, Neha Kalani A, Bachheti RK Gupta PC (2025). Comprehensive insights into leishmaniasis: From etiopathogenesis to a novel therapeutic approach. Microb Pathog.

[2] Steverding D (2017). The history of leishmaniasis. Parasit Vectors.

[3] World Health Organization (2023). Leishmaniasis.

[4] Pan American Health Organization (2024). Visceral Leishmaniasis.

[5] Alencar RB, Scarpassa VM (2018). Morphology of the eggs surface of ten Brazilian species of phlebotomine sandflies (Diptera: Psychodidae). Acta Trop.

[6] Garcia FC, Santos CFR, Santos L, Miranda PRB, Bassi ÊJ, Anderson L (2025). Entomological study of Phlebotomine Sand flies in Maceió (Brazil): 2011-2020 analysis. Braz J Biol.

[7] Bánki O, Roskov Y, Vandepitte L, DeWalt RE, Remsen D, Schalk P (2021). Catalogue of Life Checklist.

[8] Galati EAB, Rangel EF, Shaw JJ (2018). Brazilian Sand Flies: Biology, Taxonomy, Medical Importance and Control.

[9] Sousa-Paula LC, Dantas-Torres F (2021). Who is Lutzomyia longipalpis (Lutz & Neiva, 1912)?. Acta Trop.

[10] Andrade-Filho JD, Reis AS, Monteiro CC, Shimabukuro PHF (2022). Online catalogue of the Coleção de Flebotomíneos (FIOCRUZ/COLFLEB), a biological collection of American sand flies (Diptera: Psychodidae, Phlebotominae) held at Fiocruz Minas, Brazil. Gigabyte.

[11] Andrade-Filho JD, Scholte RGC, Amaral ALG, Shimabukuro PHF, Carvalho OS, Caldeira RL (2017). Occurrence and Probability Maps of Lutzomyia longipalpis and Lutzomyia cruzi (Diptera: Psychodidae: Phlebotominae) in Brazil. J Med Entomol.

[12] Almeida PS, Silva TM, Moreira RF, Mariano VF, Neto MPO, Aquino DVBS (2019). The sand fly species (Diptera: Psychodidae) in an urban environment of Mato Grosso do Sul, Brazil. J Trop Pathol.

[13] Azami-Conesa I, Gómez-Muñoz MT, Martínez-Díaz RA (2021). A Systematic Review (1990-2021) of Wild Animals Infected with Zoonotic Leishmania. Microorganisms.

[14] Aguiar Martins K, Meirelles MHA, Mota TF, Abbasi I, de Queiroz ATL, Brodskyn CI (2021). Effects of larval rearing substrates on some life-table parameters of Lutzomyia longipalpis sand flies. PLoS Negl Trop Dis.

[15] Senanayake SC, Liyanage P, Pathirage DRK, Siraj MFR, Kolitha De Silva BGDN, Karunaweera ND (2023). Impact of climatic factors on temporal variability of sand fly abundance in Sri Lanka: Longitudinal study (2018 to 2020) with two-stage hierarchical analysis. Res Sq.

[16] Ministério da Saúde (MS) (2025). TabNet - Sistemas e Aplicações.

[17] Instituto Brasileiro de Geografia e Estatística (IBGE) (2024). Unaí - MG: panorama.

[18] Pugedo H, Barata RA, França-Silva JC, Silva JC, Dias ES (2005). HP: um modelo aprimorado de armadilha luminosa de sucção para a captura de pequenos insetos. Rev Soc Bras Med Trop.

[19] Langeron M (2018). Precis de microscopie; technique, experimentation, diagnostic.

[20] Galati EAB (2024). Morfologia e terminologia de Phlebotominae (Diptera: Psychodidae). Classificação e identificação de táxons das Américas. Apostila da Disciplina Bioecologia e Identificação de Phlebotominae do Programa de Pós-Graduação em Saúde Pública.

[21] Magurran AE (2013). Ecological diversity and its measurement.

[22] Dias ES, Regina-Silva S, França-Silva JC, Paz GF, Michalsky EM, Araújo SC (2011). Eco-epidemiology of visceral leishmaniasis in the urban area of Paracatu, state of Minas Gerais, Brazil. Vet Parasitol.

[23] Capucci DC, Campos AM, Soares JVR, Ramos VDV, Binder C, Lima MA (2023). Ecology and natural infection of phlebotomine sand flies in different ecotopes and environments in the municipality of Pains, Minas Gerais, Brazil. Acta Trop.

[24] Lopes CMD, Cardoso DT, Bezerra JMT, de Araújo GR, Carneiro M, Morais MHF (2025). Spatiotemporal analysis of visceral leishmaniasis in Belo Horizonte, Brazil: a historical perspective (1994-2018). Trans R Soc Trop Med Hyg.

[25] da Silva WJ, Cardoso DT, Morais MHF, Carneiro M, Moraga P, Barbosa DS (2020). Spatiotemporal patterns and integrated approach to prioritize areas for surveillance and control of visceral leishmaniasis in a large metropolitan area in Brazil. Acta Trop.

[26] de Souza CF, Quaresma PF, Andrade JD, Bevilacqua PD (2014). Phlebotomine fauna in the urban area of Timoteo, State of Minas Gerais, Brazil. Acta Trop.

[27] Barata RA, Franca-Silva JC, Mayrink W, Silva JC, Prata A, Lorosa ES (2005). Aspects of the ecology and behaviour of phlebotomines in endemic area for visceral leishmaniasis in State of Minas Gerais. Rev Soc Bras Med Trop.

[28] Carvalho GML, Rego FD, Tanure A, Silva ACP, Dias TA, Paz GF (2017). Bloodmeal identification in field-collected sand flies from Casa Branca, Brazil, Using the cytochrome b PCR method. J Med Entomol.

[29] Brazil RP, Pontes MC, Passos WL, Rodrigues AA, Brazil BG (2011). The sand fly fauna (Psychodidae: Phlebotominae) in the region of Saquarema, State of Rio de Janeiro, Brazil, an endemic area of cutaneous leishmaniasis transmission. J Vector Ecol.

[30] Brilhante AF, Dorval ME, Galati EA, da Rocha HC, Cristaldo G, Nunes VL (2015). Phlebotomine fauna (Diptera: Psychodidae) in an area of fishing tourism in Central-Western Brazil. Rev Inst Med Trop Sao Paulo.

[31] Montes de Oca-Aguilar AC, Euan-Canul RD, Sosa-Bibiano EI, Lopez-Avila KB, Rebollar-Tellez EA, Palacio-Vargas JA (2024). Phlebotomine sand flies in rural Mayan communities of Southern Mexico: The heterogeneity of the ruralscape increases the entomological risk. Acta Trop.

[32] Hassaballa IB, Torto B, Sole CL, Tchouassi DP (2021). Exploring the influence of different habitats and their volatile chemistry in modulating sand fly population structure in a leishmaniasis endemic foci, Kenya. PLoS Negl Trop Dis.

[33] Berrozpe PE, Lamattina D, Santini MS, Araujo AV, Torrusio SE, Salomon OD (2019). Spatiotemporal dynamics of Lutzomyia longipalpis and macro-habitat characterization using satellite images in a leishmaniasis-endemic city in Argentina. Med Vet Entomol.

[34] De Oliveira EF, Silva EA, Casaril AE, Fernandes CE, Paranhos AC, Gamarra RM (2013). Behavioral aspects of Lutzomyia longipalpis (Diptera: Psychodidae) in urban area endemic for visceral leishmaniasis. J Med Entomol.

[35] Falcao de Oliveira E, Casaril AE, Fernandes WS, Ravanelli MS, Medeiros MJ, Gamarra RM (2016). Monthly distribution of phlebotomine sand flies, and biotic and abiotic factors related to their abundance, in an urban area to which visceral leishmaniasis is endemic in Corumba, Brazil. PLoS One.

[36] Guimaraes VC, Costa PL, Silva FJ, Silva KT, Silva KG, Araujo AI (2012). Phlebotomine sandflies (Diptera: Psychodidae) in Sao Vicente Ferrer, a sympatric area to cutaneous and visceral leishmaniasis in the state of Pernambuco, Brazil. Rev Soc Bras Med Trop.

[37] Barata RA, Silva JC, Costa RT, Fortes-Dias CL, Silva JC, Paula EV (2004). Phlebotomine sand flies in Porteirinha, an area of American visceral leishmaniasis transmission in the State of Minas Gerais, Brazil. Mem Inst Oswaldo Cruz.

[38] Almeida PS, Minzao ER, Minzao LD, Silva SR, Ferreira AD, Faccenda O (2010). Ecological aspects of Phlebotomines (Diptera: Psychodidae) in the urban area of Ponta Pora municipality, State of Mato Grosso do Sul, Brazil. Rev Soc Bras Med Trop.

[39] Araujo e Silva E, Andreotti R, Honer MR (2007). Behavior of Lutzomyia longipalpis, the main vector of American visceral leishmaniasis, in Campo Grande, State of Mato Grosso do Sul. Rev Soc Bras Med Trop.

[40] Rego FD, Soares RP (2021). Lutzomyia longipalpis: an update on this sand fly vector. An Acad Bras Ciênc.

[41] Leonel JAF, Vioti G, Alves ML, Spada JCP, Yamaguchi AK, Pereira NWB (2024). Species, natural Leishmania spp. detection and blood meal sources of phlebotomine sandflies (Diptera: Psychodidae: Phlebotominae) in peridomiciles from a leishmaniases endemic area of Brazil. Transbound Emerg Dis.

[42] Vieira VP, Ferreira AL, Biral dos Santos C, Leite GR, Ferreira GE, Falqueto A (2012). Peridomiciliary breeding sites of phlebotomine sand flies (Diptera: Psychodidae) in an endemic area of American cutaneous leishmaniasis in southeastern Brazil. Am J Trop Med Hyg.

[43] Souza NA, Coelho CA, Vilela ML, Peixoto AA, Rangel EF (2002). Seasonality of Lutzomyia intermedia and Lutzomyia whitmani (Diptera: Psychodidae: Phlebotominae), occurring sympatrically in area of cutaneous leishmaniasis in the State of Rio de Janeiro, Brazil. Mem Inst Oswaldo Cruz.

[44] Aguiar GM, Azevedo ACR, Medeiros WM, Alves JRC, Rendeiro V (2014). Aspects of the ecology of phlebotomines (Diptera: Psychodidae: Phlebotominae) in an area of cutaneous leishmaniasis occurrence, municipality of Angra dos Reis, coast of Rio de Janeiro State, Brazil. Rev Inst Med Trop Sao Paulo.

[45] Saraiva L, Carvalho GM, Gontijo CM, Quaresma PF, Lima AC, Falcao AL (2009). Natural infection of Lutzomyia neivai and Lutzomyia sallesi (Diptera: Psychodidae) by Leishmania infantum chagasi in Brazil. J Med Entomol.

[46] Lopes JV, Michalsky EM, Pereira NCL, de Paula AJV, Lara-Silva FO, Silva-Lana R (2019). Entomological studies in Itauna, Brazil, an area with visceral leishmaniasis transmission: fauna survey, natural Leishmania infection, and molecular characterization of the species circulating in phlebotomine sand flies (Diptera: Psychodidae). J Med Entomol.

[47] Carvalho GM, Andrade JD, Falcao AL, Rocha Lima AC, Gontijo CM (2008). Naturally infected Lutzomyia sand flies in a Leishmania-endemic area of Brazil. Vector Borne Zoonotic Dis.

[48] Lana RS, Michalsky EM, Fortes-Dias CL, Franca-Silva JC, Lara-Silva FO, Lima ACV (2015). Phlebotomine sand fly fauna and Leishmania infection in the vicinity of the Serra do Cipo National Park, a natural Brazilian heritage site. Biomed Res Int.

[49] de Oliveira AG, Andrade JD, Falcao AL, Brazil RP (2003). Study of sand flies (Diptera, Psychodidae, Phlebotominae) in the urban area of Campo Grande, Mato Grosso do Sul State, Brazil, from 1999 to 2000. Cad Saude Publica.

[50] Galati EA, Nunes VL, Rego FA, Oshiro ET, Rodrigues Chang M (1997). Estudo de flebotomíneos (Diptera: Psychodidae) em foco de leishmaniose visceral no Estado de Mato Grosso do Sul, Brasil. Rev Saúde Pública.

[51] Brazil RP, Carneiro VL, Andrade JD, Alves JCM, Falcão AL (1997). Biology of Lutzomyia lenti (Mangabeira) (Diptera: Psychodidae). An Soc Entomol Brasil.

[52] Gomes LB, Dias ES, Silva SCPF, Carvalho PCFB, Santos AGRC, Michalsky E (2019). Eco-epidemiological study on sandflies and environmental aspects related to the transmission of leishmaniasis in a municipality of Minas Gerais, Brazil, 2015-2016. Arq Bras Med Vet Zootec.

[53] de Souza Fernandes W, de Oliveira Moura Infran J, Falcao de Oliveira E, Etelvina Casaril A, Petilim Gomes Barrios S, Lopes de Oliveira SL (2022). Phlebotomine Sandfly (Diptera: Psychodidae) fauna and the association between climatic variables and the abundance of Lutzomyia longipalpis sensu lato in an intense transmission area for visceral leishmaniasis in Central Western Brazil. J Med Entomol.

[54] Cheghabaleki ZZ, Yarahmadi D, Karampour M, Shamsipour A (2019). Spatial dynamics of a phlebotomine sand flies population in response to climatic conditions in Bushehr province of Iran. Ann Glob Health.

[55] El Omari H, Chahlaoui A, Talbi FZ, Chlouchi A, El-Akhal F, Lahouiti K (2023). Entomological Survey and Impact of Climatic Factors on the Dynamics of Sandflies in Central Morocco. Scientific World Journal.

[56] Oliveira AG, Galati EA, Fernandes CE, Dorval ME, Brazil RP (2008). Seasonal variation of Lutzomyia longipalpis (Lutz & Neiva, 1912) (Diptera: Psychodidae: Phlebotominae) in endemic area of visceral leishmaniasis, Campo Grande, state of Mato Grosso do Sul, Brazil. Acta Trop.

[57] Ministério da Saúde (MS), Departamento de Vigilância Epidemiológica. Secretaria de Vigilância em Saúde (2014). Manual de Vigilância e Controle da Leishmaniose Visceral.

[58] Estallo EL, Santana M, Martin ME, Galindo LM, Willener JA, Kuruc JA (2021). Environmental effects on phlebotominae sand flies (Diptera:Phychodidae) and implications for sand fly vector disease transmission in Corrientes city, northern Argentina. An Acad Bras Cienc.

[59] Martins KA, Morais CS, Broughton SJ, Lazzari CR, Bates PA, Pereira MH (2023). Response to thermal and infection stresses in an American vector of visceral leishmaniasis. Med Vet Entomol.

[60] Virgens TM, Santos CB, Pinto IS, Silva KS, Leal FC, Falqueto A (2008). Phlebotomine sand flies (Diptera, Psychodidae) in an american tegumentary leishmaniasis transmission area in northern Espirito Santo State, Brazil. Cad Saude Publica.

[61] Condino ML, Sampaio SM, Henriques LF, Galati EA, Wanderley DM, Correa FM (1998). American cutaneous leishmaniasis: sandflies from the transmission area in the town of Teodoro Sampaio, the southeastern region of Sao Paulo state, Brazil. Rev Soc Bras Med Trop.

[62] Herrera L, Benavides-Cespedes I, Linero JD, Posada-Echeverria D, Mendoza JA, Perez-Doria A (2024). Phlebotomine sand flies (Diptera: Psychodidae, Phlebotominae): diversity of potential Leishmania vectors in northern Colombia. Acta Trop.

[63] Alexander B (2000). Sampling methods for phlebotomine sandflies. Med Vet Entomol.

[64] Lainson R, Rangel EF (2005). Lutzomyia longipalpis and the eco-epidemiology of American visceral leishmaniasis, with particular reference to Brazil: a review. Mem Inst Oswaldo Cruz.

[65] Lara-Silva FO, Michalsky EM, Fortes-Dias CL, Fiuza VOP, Dias ES (2017). Evaluation of chemical spraying and environmental management efficacy in areas with minor previous application of integrated control actions for visceral leishmaniasis in Brazil. Acta Trop.

